# Laser procedures in the treatment of BPH: a bibliometric study

**DOI:** 10.1007/s00345-020-03532-1

**Published:** 2020-12-02

**Authors:** Anja C. Reichelt, Rodrigo Suarez-Ibarrola, Thomas R. W. Herrmann, Arkadiusz Miernik, Dominik S. Schöb

**Affiliations:** 1 Department of Urology, Faculty of Medicine, University of Freiburg—Medical Center, Hugstetter Str. 55, 79106 Freiburg, Germany; 2 Department of Urology, Spital Thurgau AG (STGAG), Pfaffenholzstrasse 4, 8501 Frauenfeld, Switzerland

**Keywords:** Benign prostatic hyperplasia, Laser, Bibliometrics, Publication trends

## Abstract

**Purpose:**

To perform a bibliometric analysis of lased-based BPH treatment publications and to obtain an understanding of the publication trends over time.

**Materials and methods:**

The Medline database was searched for articles in English language regarding laser-based BPH therapy up to 2018. Matching articles were assigned to at least one of the following categories: Ho:YAG, Tm:YAG, green light, diode, Nd:YAG laser and review articles. The laser categories were analysed using bibliometric procedures regarding citation rate, authors, country of origin and journal of publication. Moreover, the articles on laser BPH therapy included in the EAU, AUA and JUA guidelines were analysed to evaluate the most influential articles.

**Results:**

In total, 982 articles were included: 317 articles were assigned to green light, 283 to Ho:YAG, 101 to Tm:YAG, 74 to Nd:YAG and 39 to diode lasers. The publication rate for Nd:YAG laser has declined, but continues to grow for Ho:YAG and Tm:YAG lasers. We found a positive correlation between number of authors and year of publication (*R* = 0.3, *p* < 0.001*). Articles on Ho:YAG lasers are cited the most (mean 23.0 ± 37.1). Asia has contributed the most articles. Mostly, countries with high health and research and development (R&D) expenditures influenced the guidelines regarding laser-based approaches. Yet, after adjustment of all search results to GDP, health and R&D expenditure, India and China were the most prolific countries.

**Conclusion:**

**L**aser-based approaches for BPH treatment are increasing but have not been implemented worldwide. Asia’s contribution should be acknowledged. An inflationary trend regarding the number of authors per article is confirmed.

## Introduction

Benign prostatic hyperplasia (BPH) is a common disease in aging men. Its prevalence is reported to be about 20% in men between 50 and 59 years old increasing to up to 40% in men over 70 [1]. BPH has also been ranked the third most common research topic in urological literature since 1955 [2]. Over the last decades, several novel surgical techniques implementing laser technology have been introduced to e.g. enucleate, resect, photovaporize the prostate or apply a combination of them. To obtain a better understanding of the research and clinical interest for various laser types in BPH therapy worldwide, we carried out a bibliometric analysis of this field.

The term *bibliometrics* refers to the “application of mathematics and statistical methods to books and other media of communication“ [3]. Applying this field of research to medical literature allows the examination of publication trends over time. In this matter, citation analyses have been carried out in surgical specialties such as orthopaedic surgery to identify articles that have provided the greatest intellectual influence [4]. Authorship trends in scientific research have been investigated and an increase in the number of authors was found throughout medical literature [5]. Moreover, the global contribution to a certain field has been examined to elucidate factors that may help create a successful research atmosphere [6].

In this study, we apply bibliometric analysis to the field of laser-based BPH therapy to analyse authorship trends, compare the scientific adoption of different laser types and investigate the characteristics of the contributing countries.

## Materials and methods

### Literature search

A full-text search was performed on 16 October 2019 in the Medline database using Web of Science. The search was limited to articles published up to 2018 since on the date of the search, complete information was not yet available for articles published in 2019. Our search strategy included a broad phrasing including the combination of, inter alia, the following terms for BPH by the term ‘OR’: “prostat* hyperplas*”, “benign prost*”, “lower urin* tract sympt*”, etc. This search was then combined with a search for laser procedures using, amongst others, the following terms: “laser enucleat*”, “photoselective vapori*”, “laser resect*”, etc.

### Inclusion and exclusion criteria

Articles meeting the following criteria were included: (a) original or review articles, (b) articles written in English language and (c) laser-based BPH therapy up to 2018. After article selection, the following types of studies were excluded: articles not written in English, editorials, conference abstracts and case reports.

### Data extraction

All articles were reviewed by title or abstract and assigned to at least one of the following categories: holmium laser (Ho:YAG), thulium laser (Tm:YAG), green light laser, diode laser, neodymium laser (Nd:YAG) and review articles. The following information was extracted from each article: title, number of authors, country of origin determined by the first author’s affiliation, journal and year of publication and number of citations.

### Bibliometric procedures

Including all years, the overall productivity over time and in each laser category was reviewed by means of the number of publications per year. The recent impact of laser procedures in the treatment of BPH in general was evaluated in terms of the top three journals publishing the majority of articles on laser-based BPH therapy each year among all urological and nephrological journals listed according to the InCites Journal Citation Report between 2010 and 2018 [7].

The number of an article’s citations, which is widely accepted as a neither entirely unproblematic nor tamper-proof bibliometric indicator [8], was analysed in each laser category by comparing the mean total citations as it provides another means to compare the importance of the different laser categories. For an adequate comparison of the different categories, citation analysis was carried out including citations from 2007 to 2018 since Tm:YAG laser articles were first published in 2007. Since group-authorship seems to increase the citation rate [9], the number of authors per article was correlated to the citation rate. To analyse authorship, the number of authors per article was evaluated and group differences were assessed.

The publication rates for each country per year and a country’s contribution to the five laser-based BPH therapeutic modalities were examined. To further analyse the contributing countries characteristics, we compared the country´s number of inhabitants in 2017 (in million) [10] to the number of publications during the period examined. We also correlated the number of publications with the latest available data for a country’s gross domestic product (GDP) [10], healthcare expenditure [11] and research and development (R&D) expenditure [12], and highlighted the most productive countries in terms of population, GDP, R&D and healthcare expenditure.

### EAU, AUA and JUA guidelines on BPH therapy

We applied the above-stated bibliometric procedures regarding country of origin, year and type of publication to articles concerning laser-based BPH therapy included in the EAU 2020 (European Urology Association) [13] and AUA 2019 (American Urology Association) [14] guidelines. Moreover, the surgical techniques addressed in the articles were considered. The UAA (Urological Association of Asia) has not yet published BPH guidelines, however, to gain an initial insight into Asian trends, we examined articles included in the JUA (Japanese Urological Association) guidelines [15]. The levels of evidence of the in the guidelines included articles were evaluated according to the levels of evidence of the Oxford Centre for Evidence-based Medicine (version March 2009) [16].

### Statistical analysis

Statistical calculations were performed using Microsoft Excel 2010 Version 14.0 (Microsoft Corp, Redmond, WA, USA). Google software was used for graphic illustrations. Descriptive statistics were used: percentages, means and standard deviation. We assessed linear univariate regressions and calculated the Pearson correlation coefficient to evaluate the distribution of the number of publications over time, the number of authors per publication per year and the citation rate throughout the number of authors. Student’s t test was used to compare differences between groups. Statistically significant group differences were considered at *p* < 0.05.

## Results

### Overall productivity

**Fig. 1:**
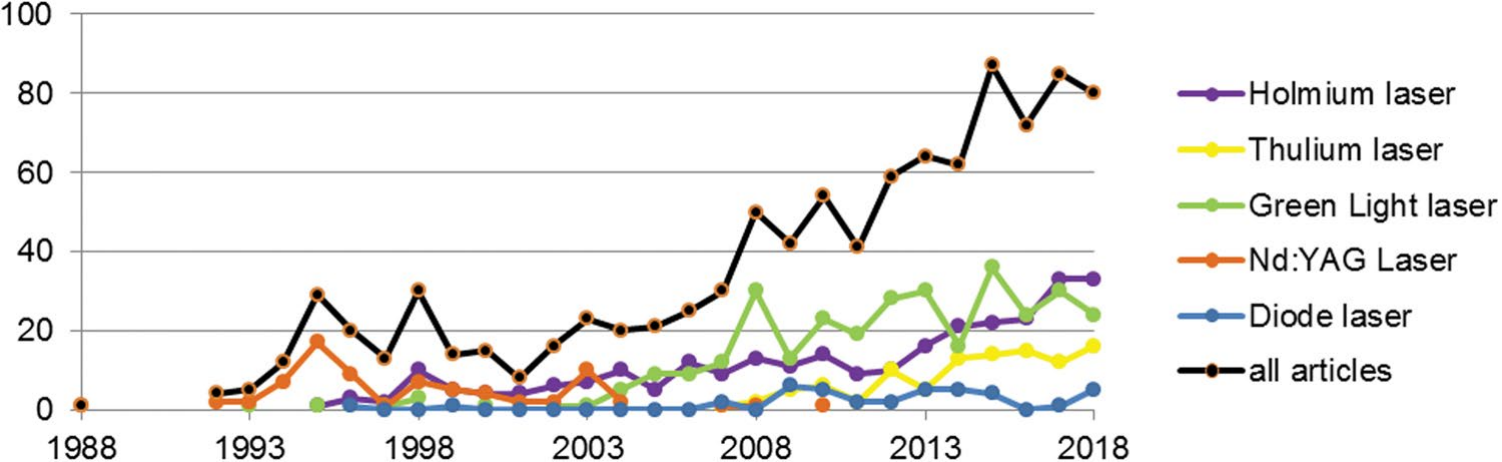
Number of articles per year, total and for laser categories

A total of 982 articles were included in our analysis. The first article was published in 1988 and addressed the Nd:YAG laser. In 2015, the overall number of publications rose to 87 articles and has since undergone a decrease (Fig. 1).

Overall, 317 articles were assigned to green light, 283 to Ho:YAG, 101 to Tm:YAG, 74 to Nd:YAG and 39 to diode lasers. The Nd:YAG category has shown a decline in its publication rate with no publications since 2010 while the diode laser’s publication rate is the lowest with a maximum of six articles per year. The green light laser category has a relatively high number of articles per year since 2007 (median: 24, range: 12–36). From 2007 to 2018, the number of Ho:YAG and Tm:YAG laser articles has increased 3.7 and 16 times, respectively.

**Fig. 2:**
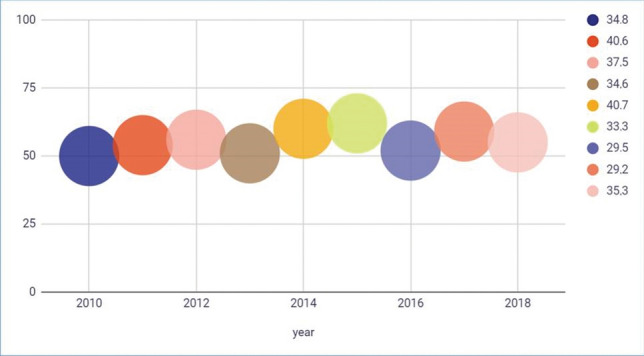
Accumulated mean position of the top three journals with the most publications regarding laser-based BPH therapy in each year among all urological and nephrological journals listed according to the InCites Journal Citation Report (0 lowest ranking–100 highest ranking). On the right, the percentage of the total number of laser articles published in that year in those top three journals is listed

The impact of laser procedures in the treatment of BPH between 2010 and 2018 was evaluated in terms of the top three journals publishing the majority of articles on laser-based BPH therapy each year (Fig. 2).

### Citations

Ho:YAG lasers articles were cited the most between 2007 and 2018 (mean 23.0 ± 37.1) and significantly more often than those concerning diode laser (mean 15.5 ± 18.6; *p* = 0.05*) and Nd:YAG laser (mean 5.4 8.2; *p* < 0.001). Nd:YAG articles were cited significantly less than all the other categories. Citations for all lasers ranged from 0 to 272 citations, the highest cited article addressing Ho:YAG laser [17]. There was no significant correlation in any category between the number of authors and citations (*p* > 0.05).

### Author’s activity

The number of authors per article was highest for Tm:YAG laser articles (6.4 ± 2.7) and significantly lower for Nd:YAG laser articles than all the other articles (4 ± 2; *p* < 0.001*). Thereby over all categories the number of authors correlated significantly to year of publication (*R* = 0.3, *p* < 0.001*).

### Countries’ activity

**Fig. 3:**
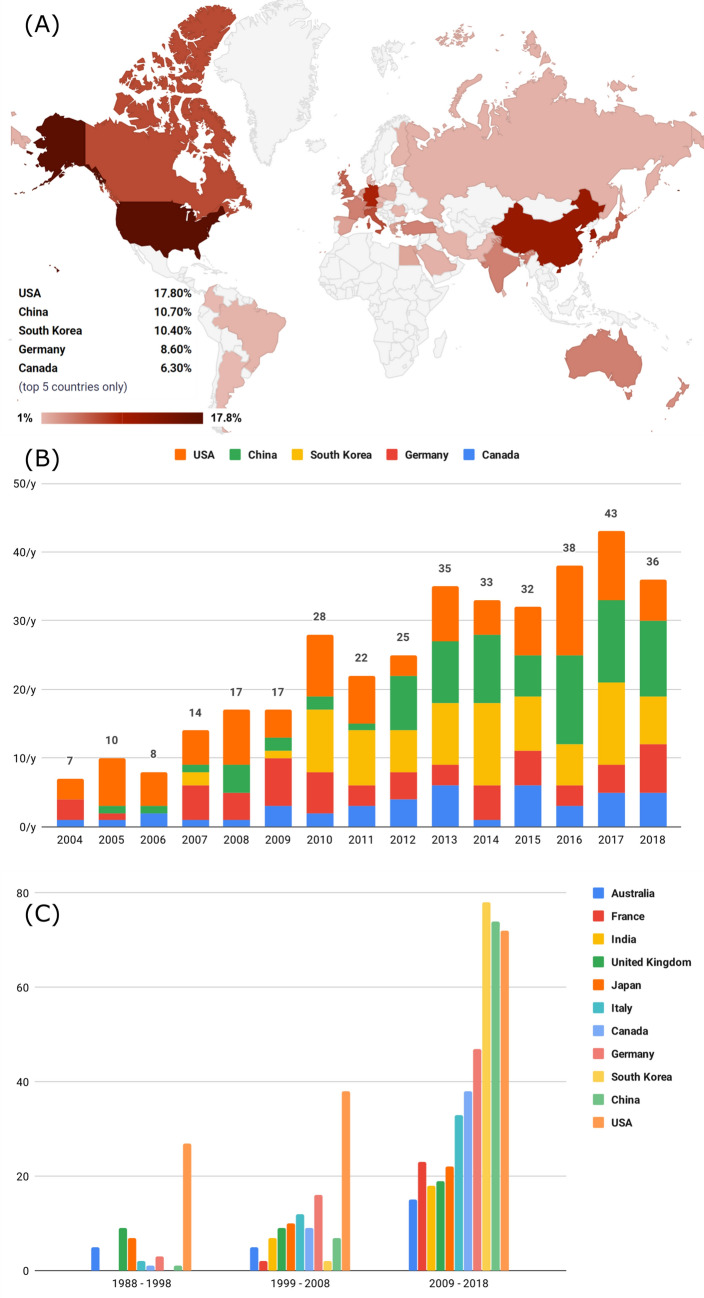
**a** Percentage shares in the global production of all contributing countries. **b** Publication rates per year for the five countries with the most publications. **c** Total number of publications in the periods from 1988 to 1998, from 1999 to 2008 and from 2009 to 2018 of the top 11 countries with the most publications regarding laser-based BPH therapy

The USA has published most of the Ho:YAG (24%) and green light laser articles (15.2%) followed by South Korea (15.2% and 11.5%, respectively). The USA also dominates the Nd:YAG category (29.6%). China published most articles regarding Tm:YAG (36%) and Germany the diode laser (18.9%). The percentage shares of the individual countries in the global contribution are illustrated on the world map in Fig. 3a. China and South Korea have only become key players in the global contribution of laser-based BPH therapy articles in the last 15 years (Fig. 3b). The development over time of the top 11 countries in terms of publication rates is illustrated in Fig. 3c. Over all five laser categories, 35.3% of the articles were published by Asian countries, 29.7% by countries of the European Union (EU), and 24.1% by North America. Particularly countries with high health expenditures and R&D expenditures have published on Ho:YAG laser (Table 1). Table 1 lists the most prolific countries in the different categories after adjusting the number of records to the above-mentioned indicators.

**Table 1:** Correlations between number of publications and demographic/economic data; most prolific countries after adjustment to demographic/economic data

1. Correlations	Green light		Holmium		Thulium		Nd:YAG		Diode	
	*r*	*P* value	*r*	*P* value	*r*	*P* value	*r*	*P* value	*r*	*P* value
Population	0.33	0.085	0.12	0.57	0.4	0.188	0.76	0.002*	0.2	0.5
GDP	0.06	0.77	0.33	0.13	-0.1	0.77	0.60	0.029*	0.18	0.55
Health expenditure	0.31	0.16	0.53	0.016*	-0.11	0.74	0.83	< 0.001*	0.06	0.54
Research and Development Expenditure	0.38	0.075	0.44	0.039*	-0.03	0.94	0.51	0.073	0.19	0.87

2. Adjustment to indicators	Green light	Holmium	Thulium	Nd:YAG	Diode
Population	Switzerland	New Zealand	Germany	Netherlands	Taiwan
GDP	China	India	China	USA	China
Health expenditure	India	India	China	USA	China
Research and Development Expenditure	India	India	China	USA	Pakistan

1. Pearson correlation coefficient (*r*) and statistical significance (*p* value) for the correlation between number of publications for each category and demographic and economic data. Significant *p*-values (< 0.05) are highlighted in bold. 2. Most prolific countries after adjustment of number of records for each category to population size, GDP, health expenditure and research and development expenditure

### EAU, AUA and JUA guidelines on BPH therapy

We found 12 reviews and 75 original articles originating from 24 countries regarding laser-based BPH therapy that are included in the EAU guidelines: 20 addressing photoselective vaporization (PVP) with green light laser, 13 holmium laser enucleation of the prostate (HoLEP), one holmium laser resection and one ablation, 11 diode laser enucleation (*n* = 6) and vaporization/ablation (*n* = 5) and 25 Tm:YAG laser resection (ThuVARP, *n* = 7) and enucleation (ThuLEP/ThuVEP; *n* = 18). All studies were published between 1995 and 2019. The Nd:YAG laser is only mentioned in one article from 1998 as a combination of Ho:YAG and Nd:YAG ablation techniques. Germany has contributed the most studies (*n* = 19) to the guidelines of which 68.4% concerned Tm:YAG laser enucleation techniques. China ranks second with 16 original articles, 56.25% equally addressing Tm:YAG laser resection and enucleation techniques. Similarly, the majority of review articles originated from China (*n* = 8). Regarding green light laser, Switzerland and USA (each *n* = 5) and Australia (*n* = 4) have contributed with the majority of articles. Canada, Italy and New Zealand have contributed the most on holmium laser (each *n* = 3) and Germany holding the highest number of cited articles (71–272 citations).

Four reviews and 34 original articles originating from 18 countries are included in the AUA guidelines: 4 addressing PVP, 17 HoLEP and 5 ThuLEP/ThuVEP and 8 ThuVARP. All studies were published between 2003 and 2017. No studies on neither the diode nor the Nd:YAG laser are included. China has contributed with the majority of original articles (n = 10), 80% concerning Tm:YAG laser, and mostly on resection techniques. Correspondingly to the EAU guidelines, only green light laser publications from Australia, Switzerland and USA are included. Eleven countries have contributed with holmium laser studies that are included in the guidelines; New Zealand ranking highest with *n* = 3, between 2007 and 2018 highly cited articles (111–179 citations).

The JUA guidelines include 12 articles, four review and eighr original articles regarding laser procedures in the treatment of BPH. One study addressed PVP and diode laser, respectively, one compared PVP to diode laser vaporization and one to HoLEP. Two studies on Tm:YAG resection techniques are included, one on ThuLEP and one comparing ThuLEP and HoLEP. Except for one European multicentre study [17], all original articles included were published by Asian countries, those regarding Tm:YAG and Ho:YAG lasers by China. The guidelines include only studies published between 2010 and 2016.

Table 2 provides an overview of the levels of evidence of the articles included in the three guidelines.

**Table 2:** Levels of evidence of the in the BPH guidelines included articles on laser-based therapy according to the Oxford Centre for Evidence-based Medicine

	Total number of articles included	1a	1b	2a	2b	3a	3b	4	5
EAU (latest update 2020)	87	12	36		16		1	21	
AUA (2018, amended 2019)	38	1	24	2	8			3	
JUA (2016)	12	4	7		1				

Level 1a: systematic review of randomised controlled trials

Level 1b: individual randomised controlled trial

Level 2a: systematic review of cohort studies

Level 2b: individual cohort study

Level 3a: systematic review of case–control studies

Level 3b: individual case–control study

Level 4: case-series

Level 5: expert opinion without explicit critical appraisal, or based on physiology, bench research or “first principles”

## Discussion

The introduction of laser technologies in recent years has enabled the development of new surgical techniques for the treatment of BPH and thus alternatives to conventional transurethral resection of the prostate. Meanwhile, bibliometric analyses have been established in Urology to examine the top cited articles [18], authorship developments [19], worldwide distribution and exchange of scientific knowledge [6, 20, 21], and citation trends in general [8]. A rising trend in the field of urological laser procedures has already been described for urolithiasis [22] and Nettleton et al. have shown a notable rise of publications on laser surgery for BPH [23]. We introduced the first bibliometric analysis thus far focusing on publication origin, authorship, journals and citations regarding laser-based BPH therapy concerning various global aspects.

### Guideline evidence

The level of evidence is high in all three guidelines. Interestingly, the AUA has based their guidelines mainly on randomised controlled trials whereas in comparison, the EAU and JUA have included stronger evidence in the form of a greater number of meta-analyses. Yet, the relatively high number of included case-series in the EAU guidelines indicates that the evidence for the application of laser-technologies in the treatment of BPH is not conclusively consolidated.

### Overall productivity

In accordance with Nettleton et al., we found a growth of scientific research on laser-based therapies for BPH [23]. Over the last decade, articles on laser procedures in the treatment of BPH were published mainly in urology and nephrology journals experiencing a slight impact factor increase from 2010 to 2015 (Fig. 2). Foreseeably, there was an increase of articles addressing Ho:YAG lasers, which can be efficiently and safely used to enucleate large prostates [24], the green light laser which shows a high hemostatic ability [25] and the Tm:YAG laser which has shown to have a better vaporization performance than the Ho:YAG laser [26]. Meanwhile, the frequency of use of the Nd:YAG laser has declined since the beginning of the twenty-first century due to complications which include transient postoperative dysuria and prolonged catheterization in up to 30% of cases for visual laser ablation [27], high retreatment rates up to 40% after 3 years for interstitial coagulation and a suitability of contact laser ablation for only small-sized prostates up to 40 ml [28]. Hybrid techniques combining green light and Nd:YAG lasers showed frequent long-term complications such as bladder neck contracture or urethral stenosis [29]. Correspondingly, the EAU, AUA and JUA guidelines do not take the Nd:YAG laser into account. Meanwhile, the diode laser has not been widely used in scientific research so far. Yet, its importance remains, at least in the European urological community, under discussion [30].

### Citations

An et al. reported that in urologic research an increase in author count is associated with increased citations [19]. However, our data did not confirm their finding. We assume that our search results included mainly single-centre studies which are mostly published by one research group only and cited less than those with a multi-centre design [31]. Yet, as all bibliometric procedures used in this analysis did not include the examination of institutional affiliations and study type, we cannot make a substantiated statement in this regard.

Holmium laser enucleation of the prostate (HoLEP) is considered, among others, a new ‘gold standard’ candidate in BPH therapy [32]. In this study, holmium laser was found to be the most cited laser treatment. Considering that holmium laser techniques were established 5–10 years earlier than the other laser types included in this study—e.g. holmium resection has been included in the EAU guidelines since 2001, HoLEP and PVP since 2011, diode laser and thulium techniques since 2014—we cannot exclude a lead time bias.

### Authors’ activity

Our analysis showed that an increase in author count over time has not only reached the field of urology in general [19] but also endourological subspecialties. With a positive correlation between the number of authors and year of publication, it is not surprising that the most recent category (Tm:YAG laser) provides the highest author count. It remains under discussion whether this finding reflects a loosening of the concept of authorship, or whether it is an expression of the increasing complexity in medical fields and interdisciplinary cooperation. Either way, an increase in the number of authors has affected the authenticity of the concept of authorship itself [33].

### Countries’ activity

It has previously been shown that the U.S. dominates the global contribution to urological research. This could be attributed to their high R&D funding and education system [6, 21]. Correspondingly, Ho:YAG laser research showed a positive correlation between a country’s number of records and their healthcare and R&D expenditure. Moreover, countries with a growing GDP are also more likely to explore technological innovations as has been shown for India [34]. In our study, this is reflected in the positive correlation between a country’s GDP and the number of records regarding the Nd:YAG laser which was the first laser type used for laser treatment of BPH and therefore paved the way for this innovative field. In addition, Henrich put forward a theory that a population exceeding a critical value may cause accumulated cultural evolution to develop toward a higher technological level [35]. This theory is confirmed by the positive correlation between the population size and Nd:YAG laser records as well.

The guideline analysis shows that Australia, Switzerland and USA, all three countries with high GDPs, healthcare and research expenditures, are the most prolific countries in terms of influencing articles on green light laser. Accordingly, laser-based BPH therapy has been stigmatized as superfluous in developing countries due to high costs of the equipment and its maintenance [36]. Therefore, the fact that laser-based BPH therapy has not yet been widely adopted worldwide can be explained by difficult accessibility due to national finances [37].

In 2015, 37.4% of articles published in urological journals were published by North America compared to 26.5% by Asia [6]. Yet, in recent years, Asia’s contribution to Urology has notably increased [20] and more articles on the different laser approaches originated from Asia than from North America. While South Korea is an important participant in research on Ho:YAG and green light laser, we showed that China and India should be acknowledged as important participants despite much lower health and R&D expenditure than other countries.

Due to low operational costs and a large number of healthy volunteers, India is considered one of the most preferred places for clinical trials [38]. Therefore, their potential in future research in laser-based BPH therapy should not be underestimated.

All examined guidelines acknowledge China as a key player on Tm:YAG laser research. In fact, the Tm:YAG was first introduced in China [39] and according to UNESCO data, despite the low funding, China's human resources in R&D far exceed those of other countries [12]. Yet, the EAU and AUA guidelines analyses for the management of BPH has shown that China’s impact on research on Tm:YAG is mainly restricted to vaporesection techniques. Studies have shown that retreatment rates for ThuVARP might be significantly higher than for enucleation techniques and that this approach is not suitable for small prostates due to the high rate of bladder neck stenosis [40, 41]. Therefore, the current evidence indicates a superiority of ThuLEP/ThuVEP, to which at least in terms of the EAU guidelines, Germany has shown the most notable contribution.

This study is not devoid of limitations. Although a comprehensive search was carried out, restrictions to the Medline database and to articles written in English might have failed to retrieve all articles relevant to the topic. Furthermore, we limited our analysis of country activity to the first author´s affiliation. A more convincing statement about the world’s contribution in this regard may be achieved by further analyzing the country of origin of all authors in each publication. Unfortunately, for population size and economic factors, the latest data available referred to 2017 or earlier and the countries’ developments in those fields should also be included. Moreover, we mainly presented a quantitative analysis. Further analyses would be desirable regarding the qualitative contribution of different countries, journals and authors through their citation index as well as their positive and negative growth over time.

## Conclusion

The bibliometric methods employed in this article provide a comprehensive approach to follow and summarize the development of highly dynamic fields such as laser-based BPH treatment and help to better understand the driving forces in a global context. Although the Ho:YAG laser is the highest cited category, it is still a treatment modality requiring a high level of expertise. Mainly countries with high R&D and healthcare expenditure influence the recommendations on laser technologies in BPH therapy. Yet, our findings indicate that R&D and healthcare expenditure by themselves are not significant predictors for the quantity and quality of a country's research.

The Nd:YAG laser is the only laser type whose relevance has declined. The conduction of more clinical trials and Asian countries as the main contributors regarding other laser types is a probable scenario. Furthermore, journal editors should note that an inflationary increase in author count has reached the field of laser-based BPH therapy.

## Data Availability

Data available on request from the authors.
